# Draft genomes of two *Enterobacter hormaechei* strains isolated from catheterized urine samples from females experiencing overactive bladder symptoms

**DOI:** 10.1128/mra.00491-24

**Published:** 2024-07-16

**Authors:** Helen Appleberry, Richa Patel, Kanishka Singh, Alan J. Wolfe, Catherine Putonti, Alex Kula

**Affiliations:** 1 Department of Biology, Loyola University Chicago, Chicago, Illinois, USA; 2 Bioinformatics Program, Loyola University Chicago, Chicago, Illinois, USA; 3 Department of Microbiology and Immunology, Loyola University Chicago, Maywood, Illinois, USA; Department of Biological Sciences, Wellesley College, Wellesley, Massachusetts, USA

**Keywords:** *Enterobacter hormaechei*, urinary microbiome, OAB

## Abstract

In this study, we present the draft genome of two *Enterobacter hormaechei* strains isolated from catheterized urine specimens from females with overactive bladder (OAB) symptoms. Through the sequencing of these *E. hormaechei* strains, we aim to better understand its presence and putative role in OAB in the female urinary tract.

## ANNOUNCEMENT

*Enterobacter hormaechei,* a member of the *Enterobacter cloacae* complex (ECC), is a prominent pathogen frequently identified in clinical settings due to its prevalence in diverse sample types (1). Associated with a broad spectrum of nosocomial infectious diseases such as bacteremia, biliary tract infections, and urinary tract infections (UTIs), *E. hormaechei* poses a significant risk in clinical management as it exhibits a concerning level of resistance to numerous antibiotics (1, 2). In this study, we present the genome sequencing of two *E. hormaechei* strains, isolated from catheterized urine samples from two different females with overactive bladder (OAB) symptoms (3). Although *E. hormaechei* is the most frequent species related to *Enterobacter*-associated UTI (4), an association between this species and OAB has not yet been suggested.

Urine samples were collected as part of a prior institutional review board-approved study (LU207152) (3). Using the enhanced quantitative urine culture (EQUC) method (5), each strain was isolated, and the species identification was made via MALDI-TOF, as previously described (6). Each isolate was then stored in the Loyola Urinary Education and Research Collaborative (LUEREC) collection at −80°C. From the collection, we obtained Columbia Colistin Naladixic Acid (CNA) plates with the strains. Individual colonies were selected and streaked onto nutrient broth (NB) agar plates and incubated in 5% CO_2_ at 35°C for 24 hours. A single colony was selected and grown in NB medium in 5% CO_2_ at 35°C for 24 hours. DNA extraction was carried out using the Qiagen DNeasy Blood and Tissue Kit according to the manufacturer’s instructions for Gram-positive bacteria. DNA was quantified using a Qubit fluorometer. Library preparation and sequencing were performed by SeqCoast Genomics (Portsmouth, NH). Libraries were constructed using the Illumina DNA Prep tagmentation kit and unique dual indexes following the manufacturer’s protocol. Using a 300-cycle flow cell kit, sequencing was carried out on the Illumina NextSeq2000 platform (2 × 150 bp). Using the assembly service (“auto” parameter) through the website BV-BRC v3.35.5 (7), reads were trimmed by trim_galore v0.6.5dev (https://github.com/FelixKrueger/TrimGalore) and assembled with Unicycler v0.4.8 (8). Assemblies were polished in two rounds using pilon v1.23 (9). The genome was annotated using the NCBI Prokaryotic Genome Annotation Pipeline (PGAP) v6.7 (10) upon submission. Genome coverage, completeness, and contamination were calculated by BV-BRC and NCBI upon submission. Antibiotic resistance genes were identified using ResFinder v4.5.0 (11, 12), specifying the “Other” species option. Unless otherwise stated, default parameters were used for all software tools.

Information about the genome assemblies for *E. hormaechei* UMB2909 and UMB4920 can be found in Table 1. Both strains’ genomes were examined for antibiotic resistance genes, identifying *blaACT*, associated with beta-lactam resistance, and *fosA*, associated with fosfomycin resistance. The two strains had identical copies of these genes having 99.48% and 97.18% sequence identity to the resistance genes in the ResFinder database, respectively. In a prior study in 2001, *E. hormaechei* was identified as the member of ECC that was most susceptible to fosfomycin (13); future studies on this resistance among current strains in circulation must be carried out to assess if this resistance has increased over time or if simply the two strains examined here are outliers.

**TABLE 1:** Genome assembly statistics for *E. hormaechei* UMB2909 and UMB4920

Strain	UMB2909	UMB4920
No. of raw reads	2,665,950	1,801,394
Assembly length (bp)	4,888,222	4,888,358
G + C (%)	55.48	55.48
No. of contigs	37	38
Contigs N50 (bp)	396,383	396,383
Coverage (x)	73.73	49.35
Completeness (%)	99.51	99.51
Contamination (%)	1.19	1.19

## Data Availability

Raw reads are publicly available in the SRA database under accession nos SRR28710904 (UMB2909) and SRR28710901 (UMB4920). This Whole Genome Shotgun project has been deposited in DDBJ/ENA/GenBank under the accession nos JBCGEH000000000 (UMB2909) and JBCGEG000000000 (UMB4920). The versions described in this paper are the first version.

## References

[1] Davin-Regli A , Lavigne J-P , Pagès J-M . 2019. Enterobacter spp.: update on taxonomy, clinical aspects, and emerging antimicrobial resistance. Clin Microbiol Rev 32:e00002-19. doi:10.1128/CMR.00002-19 31315895 PMC6750132

[2] Martins ER , Bueno MFC , Francisco GR , Casella T , de Oliveira Garcia D , Cerdeira LT , Gerber AL , de Almeida LGP , Lincopan N , de Vasconcelos ATR , Nogueira MCL , Estofolete CF . 2020. Genome and plasmid context of two rmtG-carrying Enterobacter hormaechei isolated from urinary tract infections in Brazil. J Glob Antimicrob Resist 20:36–40. doi:10.1016/j.jgar.2019.06.020 31279132

[3] Thomas-White K , Taege S , Limeira R , Brincat C , Joyce C , Hilt EE , Mac-Daniel L , Radek KA , Brubaker L , Mueller ER , Wolfe AJ . 2020. Vaginal estrogen therapy is associated with increased Lactobacillus in the urine of postmenopausal women with overactive bladder symptoms. Am J Obstet Gynecol 223:727. doi:10.1016/j.ajog.2020.08.006 PMC7609597 32791124

[4] Akbari M , Bakhshi B , Najar Peerayeh S . 2016. Particular distribution of Enterobacter cloacae strains isolated from urinary tract infection within clonal complexes. Iran Biomed J 20:49–55. doi:10.7508/ibj.2016.01.007 26498349 PMC4686808

[5] Price TK , Dune T , Hilt EE , Thomas-White KJ , Kliethermes S , Brincat C , Brubaker L , Wolfe AJ , Mueller ER , Schreckenberger PC . 2016. The clinical urine culture: enhanced techniques improve detection of clinically relevant microorganisms. J Clin Microbiol 54:1216–1222. doi:10.1128/JCM.00044-16 26962083 PMC4844725

[6] Hilt EE , McKinley K , Pearce MM , Rosenfeld AB , Zilliox MJ , Mueller ER , Brubaker L , Gai X , Wolfe AJ , Schreckenberger PC . 2014. Urine is not sterile: use of enhanced urine culture techniques to detect resident bacterial Flora in the adult female bladder. J Clin Microbiol 52:871–876. doi:10.1128/JCM.02876-13 24371246 PMC3957746

[7] Olson RD , Assaf R , Brettin T , Conrad N , Cucinell C , Davis JJ , Dempsey DM , Dickerman A , Dietrich EM , Kenyon RW , et al. . 2023. Introducing the bacterial and viral bioinformatics resource center (BV-BRC): a resource combining PATRIC, IRD and ViPR. Nucleic Acids Res 51:D678–D689. doi:10.1093/nar/gkac1003 36350631 PMC9825582

[8] Wick RR , Judd LM , Gorrie CL , Holt KE . 2017. Unicycler: resolving bacterial genome assemblies from short and long sequencing reads. PLoS Comput Biol 13:e1005595. doi:10.1371/journal.pcbi.1005595 28594827 PMC5481147

[9] Walker BJ , Abeel T , Shea T , Priest M , Abouelliel A , Sakthikumar S , Cuomo CA , Zeng Q , Wortman J , Young SK , Earl AM . 2014. Pilon: an integrated tool for comprehensive microbial variant detection and genome assembly improvement. PLoS One 9:e112963. doi:10.1371/journal.pone.0112963 25409509 PMC4237348

[10] Tatusova T , DiCuccio M , Badretdin A , Chetvernin V , Nawrocki EP , Zaslavsky L , Lomsadze A , Pruitt KD , Borodovsky M , Ostell J . 2016. NCBI prokaryotic genome annotation pipeline. Nucleic Acids Res 44:6614–6624. doi:10.1093/nar/gkw569 27342282 PMC5001611

[11] Bortolaia V , Kaas RS , Ruppe E , Roberts MC , Schwarz S , Cattoir V , Philippon A , Allesoe RL , Rebelo AR , Florensa AF , et al. . 2020. ResFinder 4.0 for predictions of phenotypes from genotypes. J Antimicrob Chemother 75:3491–3500. doi:10.1093/jac/dkaa345 32780112 PMC7662176

[12] Camacho C , Coulouris G , Avagyan V , Ma N , Papadopoulos J , Bealer K , Madden TL . 2009. BLAST+: architecture and applications. BMC Bioinformatics 10:421. doi:10.1186/1471-2105-10-421 20003500 PMC2803857

[13] Stock I , Grüger T , Wiedemann B . 2001. Natural antibiotic susceptibility of strains of the Enterobacter cloacae complex. Int J Antimicrob Agents 18:537–545. doi:10.1016/s0924-8579(01)00463-0 11738341

